# Applying the Integrated Sustainability Framework to explore the long-term sustainability of nutrition education programmes in schools: a systematic review

**DOI:** 10.1017/S1368980023001647

**Published:** 2023-10

**Authors:** Leila Isabella Fathi, Jacqueline Walker, Clare Frances Dix, Jessica Rose Cartwright, Suné Joubert, Kerri Amelia Carmichael, Yu-Shan Huang, Robyn Littlewood, Helen Truby

**Affiliations:** 1 School of Human Movement and Nutrition Sciences, The University of Queensland, St Lucia, QLD 4067, Australia; 2 Health and Wellbeing Queensland, Brisbane, Australia

**Keywords:** Child, Pediatric obesity, Health promotion, Implementation science, Sustainability

## Abstract

**Objective:**

This review aimed to identify and synthesise the enablers and barriers that influence the long-term (≥ 2 years) sustainment of school-based nutrition programmes.

**Design:**

Four databases (PubMed, Cochrane Library, Embase and Scopus) were searched to identify studies reporting on the international literature relating to food and nutrition programmes aimed at school-age (5–14 years) children that had been running for ≥ 2 years (combined intervention and follow-up period). Eligible studies were analysed using the Integrated Sustainability Framework (ISF), which involved deductive coding of programme enablers and barriers. A quality assessment was completed, using the Mixed-Methods Appraisal Tool and the Preferred Reporting Items for Systematic Reviews and Meta-Analyses guidelines.

**Setting:**

International school-based nutrition programmes.

**Subjects:**

Individuals involved with the implementation of school-based nutrition programmes.

**Results:**

From the 7366 articles identified, thirteen studies (seven qualitative, five mixed methods and one quantitative descriptive) were included, from which the enablers and barriers of eleven different nutrition-related programmes were analysed. Thirty-four factors across the five domains of the ISF were identified that influenced the sustained implementation of programmes. The most common barrier was a lack of organisational readiness and resources, whereas the most common enabler was having adequate external partnerships and a supportive environment.

**Conclusions:**

These findings have application during the initiation and implementation phases of school-based nutrition programmes. Paying attention to the ‘outer contextual factors’ of the ISF including the establishment and maintenance of robust relationships across whole of government systems, local institutions and funding bodies are crucial for programme sustainment.

The pervasiveness of childhood obesity has been recognised as a global public health issue. The WHO has reported that in the 40 years leading up to 2016, the number of children and adolescents with obesity had increased more than 10-fold, from 11 million to 124 million^(1)^. Further to this, WHO estimated that 216 million children and adolescents had overweight, but not obesity^(1)^. The global economic impacts of children living with obesity are estimated at (USD) $2 trillion, which is a similar economic impact to that of smoking^(2)^. Evidence suggests lifestyle, behavioural and eating habits adopted during childhood can contribute to lifelong health maintenance and, thus, reduce the risk of chronic disease onset^(3–5)^. Effective and sustained healthy lifestyle interventions during childhood are therefore required.

Schools are an ideal setting for implementing comprehensive interventions which include environmental modifications and have been utilised in many countries^(1)^. A major contributing factor to the effectiveness of school-based settings for health promotion interventions is the ability to advocate for healthy behaviours at a population level, reaching children of diverse ethnic and socio-economic backgrounds, their family members, school staff and participating community members^(5–7)^. Schools offer a unique setting whereby learning and personal development are key objectives of daily activities^(8)^. This presents an ideal setting to nurture and reinforce healthy behaviours to cultivate lifelong healthy food habits from a young age^(8)^.

In recent years, there has been continuous efforts to use schools as setting for health promotion interventions around food behaviours such as eating more fruit and vegetables. However, there remains a dearth of information regarding how to implement and sustain an effective programme beyond the duration of its funding^(9)^. Despite several systematic literature reviews reporting on and summarising data relating to the effectiveness and efficacy of school-based interventions^(9,10)^, there has been little attention directed at identifying key enablers and barriers which are directly related to long-term sustainability of any intervention in the school setting. For the context of this review, sustainability has been defined based on the review by Moore *et al*.^(11)^: (i) after a defined period of time, (ii) a programme or implementation strategy continue to be delivered and/or maintained; (iii) the programme may evolve or adapt while (iv) continuing to produce benefits for individuals/systems.

Most school-based programmes are abandoned within 2 years of commencement particularly after the withdrawal of start-up funding or resources^(9,12,13)^. Chaudhary *et al*.^(9)^ reported on short-term nutrition interventions (*n* 19), with a duration of 1 year or less, which showed that multi-component interventions can be effective in promoting healthy dietary behaviour, attitudes towards food and anthropometry, among young children. However, there was a significant decline in the number of programmes that are conducted beyond this time frame and no exploration on the long-term enablers or barriers to sustainable implementation^(9)^. A 2013 meta-analysis on the effectiveness of school-based interventions in reducing childhood obesity concluded from their meta-regression of thirty-two studies that long-term intervention lasting 1–4 years was more effective than shorter ones^(7)^. However, no studies had an intervention (including follow-up period) that surpassed 4 years^(7)^. Programmes and their core components are rarely sustained in their entirety, and examples of sustainable programmes are scarce past the 1–2-year time frame^(12)^. If effective programmes are discontinued, investments of time, people and resources cannot be optimised, which can result in loss of trust within communities, and not support the long-term health benefits for participants or economic benefits to be achieved^(12,14)^. This implies an incompleteness within current literature and has been recognised as an area requiring further exploration^(8)^.

Shoesmith *et al*.^(15)^ reviewed enablers and barriers that influence the sustainability of interventions that address risk factors for chronic diseases in the school and childcare setting^(11)^. Studies were considered eligible if external support to intervention implementation had been ceased at least 6 months prior to follow-up data collection. However, a minimum time period for programme implementation was not specified in their inclusion criteria^(15)^. Results were collated using the Integrated Sustainability Framework (ISF) and showed that factors that related to the ‘inner contextual factors’ of an organisation, such as availability of facilities or equipment, executive or leadership support and team cohesion, were essential for intervention sustainability^(15)^.

Gaining a comprehensive understanding of the enablers and barriers that affect sustainability is important to inform the planning process at the outset, including programme development, delivery and ensuring that a long-term vision for the programme to continue is enabled from the outset. This can ensure that sustainability is embedded within programme initiation and that strategies are developed that specifically identify priority determinants of long-term sustainability^(15)^. This review aims to fill some gaps by identifying and synthesising the enablers and barriers that influence the sustained implementation (≥ 2 years duration) of school-based nutrition programmes (programmes with nutrition as a key focus) for children aged between 5 and 14 years.

## Methods

This review was developed using the Preferred Reporting Items for Systematic Review and Meta-Analyses framework^(16)^. The protocol for the review was not registered.

### Information sources and search strategy

Four databases were searched for eligible studies (PubMed, Cochrane Library, Embase and Scopus), using the search strategy in online Supplementary Material 1. The searches were undertaken on 4th March 2021 by four authors (JC, KC, YH, SJ) and confirmed by another author (LF). The Problem, Intervention, Comparison, Outcome (PICO) format was followed to create a searchable question that was not formally validated but was peer reviewed by the author team (HT, JW) in collaboration with a university librarian^(17)^. A recent review paper was consulted to ensure specific and relevant search terms were captured and to support the comprehensiveness of the search strategy^(9)^. The following MeSH terms were utilised: ‘students’, ‘child’, ‘adolescent’, ‘health promotion’, ‘schools’, ‘dietetics’, ‘diet’, ‘programme evaluation’. Papers containing the word ‘adult’, without mention of ‘child’ or ‘children’, were not retrieved from databases for screening. The search and MeSH terms were developed for PubMed and then adjusted using SR-Accelerator polyglot for Cochrane Library and Embase compatibility^(18)^. The Scopus translation was completed manually. These search strings can be found in online Supplementary Material 1.

### Eligibility criteria

#### Inclusion

Qualitative, quantitative and mixed-methods studies were included for completeness provided they were peer reviewed and published in the English language. The inclusion criteria were that the studies reported on school-based health programmes which included nutrition education that aimed to promote dietary behaviour change in children. The children had to either be aged 5–14 years, in primary/elementary school or middle school, or described as adolescent^(9)^. The combined intervention and follow-up period had to be ≥ 2 years in duration, and that the intervention was included in school curricula and run during school hours. Results had to report on enablers and barriers to programme implementation and/or sustainability.

#### Exclusion

Systematic review papers; grey literature; study protocols; studies not reporting primary outcomes; or supplementary material for conferences/journals were deemed ineligible. Studies were excluded if the reported programme outcomes were primarily targeted at children, adolescents or adults outside the age range of 5–14 years old. Interventions consisting only of school meal/food/supplement provision (including canteen and free fruit and vegetable programmes) or school/community gardening programmes without nutrition education were excluded, as well as interventions aiming to prevent/overcome malnutrition or food insecurity. Studies where the primary outcome was a result of home-based, before- or after-school interventions were also excluded.

### Selection process and data collection

Eligible papers were exported to Covidence, an online software that enables multiple authors to screen through papers^(19)^. All duplicate papers were removed. Six authors (JC, KC, YH, SJ, LF and HT) screened the titles and abstracts of eligible papers. This process required consensus between two reviewers, with a third author (LF) resolving any conflicting votes. The full text of included papers was then screened by two authors (LF and JW) with a third (HT) resolving conflicting votes.

### Data extraction

Key study characteristics were extracted and transferred into a standardised Excel table by two authors (LF and JW) (Tables 1 and 2), which related to eleven different interventions (programmes). Enablers and barriers of sustained implementation were summarised and described in online Supplementary Material 3. Data were categorised according to programme titles to focus on characteristics supporting long-term implementation. Any discrepancies in data extraction were resolved by reaching consensus or by a third author (HT).

**Table 1:** Study characteristics and aims of all included studies

Author	Year published	Country	Study design	Study title	Study aims	Programme title	Programme aim
Biggs J., *et al*.^(26)^	2014	Australia	Qualitative	Applying process mapping and analysis as a quality improvement strategy to increase the adoption of fruit, vegetable, and water breaks in Australian primary schools	To provide a practical example of the use of process mapping and analysis to improve the quality of Crunch&Sip	Crunch&Sip	To increase children’s intake of fruits and vegetables
Calder K., *et al*.^(22)^	2017	New Zealand	Mixed methods	Education setting-based health promotion in New Zealand: evaluating the wellbeing and vitality in education (WAVE) programme	To report on findings from the process evaluation carried out during WAVE’s first 5 years of implementation and the findings from the impact evaluation. To describe the context of the implementation of the WAVE programme	Wellbeing and vitality in education (WAVE)	To have comprehensive promotion of health in schools, through recognising the opportunity to improve health through the education setting
Friend S., *et al*.^(23)^	2014	USA	Mixed methods	The researchers have left the building: what contributes to sustaining school-based interventions following the conclusion of formal research support?	To explore and understand the process of sustaining New Moves, including identification of outcome and potential facilitators and barriers to sustaining a school-based intervention successfully	New Moves	To decrease weight-related problems in adolescent girls
Gittelsohn J., *et al*.^(24)^	2003	USA	Mixed methods	School climate and implementation of the Pathways study	To examine support and barriers for Pathways	Pathways	To prevent obesity in American Indian school children by encouraging healthy eating and physical activity
Greaney M., *et al*.^(33)^	2014	USA	Qualitative	Implementing a multi-component school-based obesity prevention intervention: a qualitative study	To explore barriers and facilitators to implementing and sustaining Healthy Choices	Healthy Choices	To increase physical activity and healthful eating and to decrease television viewing, with the goal of reducing overweight and obesity
Hayes C., *et al*.^(31)^	2019	Ireland	Qualitative	Barriers and facilitators to adoption, implementation and sustainment of obesity prevention interventions in schoolchildren: a DEDIPAC case study	To explore the implementation of Food Dudes (barriers and facilitators to adoption, implementation and sustainability)	Food Dudes	To encourage primary school children to consume more fruit and vegetables
McIsaac J., *et al*.^(30)^	2015	Canada	Qualitative	Applying theoretical components to the implementation of Health-Promoting Schools	To describe a provincial case study of Health-Promoting Schools implementation using theoretical components	Health-Promoting Schools (HPS)	To support physical activity and healthy eating strategies across schools using a comprehensive approach
Middleton G., *et al*.^(32)^	2012	England	Qualitative	A qualitative exploration of stakeholder perspectives on a school-based multi-component health promotion nutrition programme	To investigate the receipt and delivery of the Food for Fitness programme, as perceived by local stakeholders who had experienced and administered the service	Food for Fitness	To promote healthier eating practices for children by developing food knowledge, food skills, self-confidence/self-esteem and providing specialist advice on school services for catering
Nathan N., *et al*.^(28)^	2017	Australia	Quantitative (descriptive)	Factors associated with the implementation of a vegetable and fruit programme in a population of Australian elementary schools	To identify factors associated with the implementation of a school vegetable and fruit programme	Crunch&Sip	To increase children’s intake of fruits and vegetables
Naylor P., *et al*.^(25)^	2010	Canada	Mixed methods	Implementing a whole school physical activity and healthy eating model in rural and remote First Nations schools: a process evaluation of Action Schools!BC	To explore the feasibility and implementation of AS! BC in three remote Aboriginal communities in northern British Columbia	Action Schools! BC	To enhance healthy eating and physical activity opportunities for children
Naylor P., *et al*.^(29)^	2016	Canada	Mixed methods	A mixed-methods exploration of implementation of a comprehensive school healthy eating model one year after scale-up	To study the implementation of a school-based healthy eating model one year after scale-up in British Columbia	Action Schools! BC	To enhance healthy eating and physical activity opportunities for children
Phaitrakoon J., *et al*.^(34)^	2014	Thailand	Qualitative	The Diamond Level Health-Promoting Schools (DLHPS) programme for reduced child obesity in Thailand: lessons learned from interviews and focus groups	To review and analyse the existing obesity management programmes of DLHPS and document lessons learned from these programmes to inform guidelines	The Diamond Level Health-Promoting Schools (DLHPS)	To improve students’ health through sustainable health promotion and strengthening weight control policy and programmes
Verjans-Janssen S., *et al*.^(27)^	2020	Netherlands	Qualitative	Implementation of KEIGAAF in primary schools: A Mutual Adaptation Physical Activity and Nutrition Intervention	To evaluate the implementation and contextual factors affecting implementation of the programme in primary schools	Kansen in Eindhoven voor GezinsAAnpak met Fontys (KEIGAAF). ‘Chances in Eindhoven for a family-based approach by Fontys’	To create a school environment that stimulates children to be active and have healthy eating behaviours

**Table 2:** Study characteristics and findings of included studies

Author	Programme components	Programme participants	Intervention duration (at time of reporting)	Study participants	Findings
Biggs J., *et al*.^(26)^	To provide a time in class for children to consume a piece of vegetable or fruit they have brought from home, and to drink water	Primary school children. Ages not specified	3 years	Local Health District health promotion officers and a programme coordinator	The process of delivering the programme to schools should be simplified and streamlined. Monitoring and feedback loops to track ongoing participation should also be introduced
Calder K., *et al*.^(22)^	Follows the Health-Promoting Schools (HPS) model. Working in partnership, focusing on the school food environment; involving children, parents, Maori and the community	Early childhood, primary and secondary schools and tertiary providers. Ages not specified	5 years	Programme implementers	A partnership between health and education sectors can provide the basis for high levels of participation and significant changes in practice across all levels of education and a whole province
Friend S., *et al*.^(23)^	The programme had multiple areas of focus: (1) one semester of an all-girls PE; (2) classroom sessions that focused on nutrition and social support modules taught one day/week and (3) maintenance activities outside of class including periodic individual counselling sessions and weekly lunch get-togethers in the semester	Adolescent girls. Ages not specified	2 years	PE teachers currently teaching the programme	Programmes are most likely to be sustained if they: (1) fit into the current school structure; (2) receive buy-in by teachers and (3) require minimal additional funds or staff time
Gittelsohn J., *et al*.^(24)^	Interventions across the classroom curriculum, food service, physical activity and family	Elementary school children (third to fifth grades). Ages not specified	3 years	School administrators, food service managers, classroom teachers and physical education instructors	School administration and lack of family participation were perceived barriers at some schools. A positive school climate was supported by having a classroom curriculum on healthy eating and physical activity
Greaney M., *et al*.^(33)^	The programme had multiple areas of focus: (1) have a teacher in each core subject area to teach Planet Health lessons, (2) implement a before- or after-school programme focused on nutrition or physical activity each year, (3) implement a campaign promoting the 5–2– 1 message, (4) complete a module of the School Health and (5) initiate a policy or environmental change to support healthy eating and/or active living	Middle school girls. Ages not specified	3 years	Middle school employees (administrators, teachers, food service personnel and employees serving as intervention coordinators)	State-mandated testing, budget limitations and time constraints were viewed as implementation barriers, whereas staff buy-in, external support and technical assistance were seen as facilitating implementation. Respondents thought that intervention sustainability depended on external funding and expert assistance
Hayes C., *et al*.^(31)^	Peer modelling and rewards-based intervention to increase fruit and vegetable consumption	Primary school children. Ages not specified	10 years	Major stakeholders (funders, intermediaries), teachers, academic researcher	Supportive working relationships within and across government departments, intermediaries and schools were critical for intervention successful implementation and sustainability. Organisational and leadership abilities of coordinators were essential. Successful implementation was hindered by funding insecurity, timetable constraints, lack of specificity of programme components. Supportive actions for maintenance were ongoing political support, secure funding and pre-existing healthy lifestyle policies.
McIsaac J., *et al*.^(30)^	The focus was on the following areas: developing local policy, achieving administrative support, assessing needs and developing a plan to achieve goals	School children aged 10–11 years	8 years	Principals, parents, teachers, community volunteers	Higher level visioning and school-level leadership were critical in sustaining the adoption and implementation of HPS across schools and enabled the integration into organisational processes
Middleton G., *et al*.^(32)^	Interventions were designed to promote changes in the school environment across the following areas: curriculum development, policy formation and increasing the accessibility for healthy food choices	Children in primary and secondary school settings. Ages not specified	3 years	Stakeholders (health professionals, teachers, senior health officers)	Stakeholders’ main concern was the limited capacity and size of the service. Problems with long-term sustainability in supporting schools were about lack of support and poor planning and organisation of interventions
Nathan N., *et al*.^(28)^	To provide a time in class for children to consume a piece of vegetable or fruit they have brought from home	Elementary school children. Ages not specified	9 years	School principals	Schools were significantly more likely to implement the programme if the principal believed that: the programme was effective; they had sufficient resources to implement the programme; the programme would not be difficult to implement and that the programme was as important as other school priorities
Naylor P., *et al*.^(25)^	The model targets six key ‘zones’ for action: (i) the environment (including policies); (ii) the classroom; (iii) physical education; (iv) extra-curricular; (v) school spirit and (vi) family and community	Elementary school children. Ages not specified	4 years	School principals and teachers	Implementation facilitators were having school champions, technical support and access to resources. Barriers were lack of time, loss of leadership or momentum
Naylor P., *et al*.^(29)^	Six action areas to address physical activity and healthy eating. These include: (1) school environment, (2) scheduled physical education, (3) classroom action, (4) family and community, (5) extra-curricular and (6) school spirit	Elementary school children. Ages not specified	5 years	School teachers and administrators	Support from the AS! BC head trainer and support team was crucial to the delivery of the programme. Staff highlighted challenges (e.g. lack of time, high staff turnover, lack of financial resources); however, with continued support and cultural adaptations they would continue to implement the programme
Phaitrakoon J., *et al*.^(34)^	HPS consists of 10 elements for assessment and implementation: (1) school policy, (2) management in the school, (3) collaboration of school and community, (4) creating environments supportive of health, (5) school health services, (6) health education in school, (7) nutrition and safety of food at school, (8) exercise through sport and recreation, (9) provision of counselling and social support and (10) health promotion for school staff	School children. The average age was 10·7 ± 1·1 years and most were 12 years old	3 years	School directors, teachers, cooks, students	Teamwork has been a key strategy in programme implementation. Greatest success factor was intersectoral cooperation. Challenges included confusion about the criteria for obtaining the DLHPS status, lack of parental involvement and students’ resistance to consume vegetables and other healthy foods
Verjans-Janssen S., *et al*.^(27)^	Each participating school forms a working group. The working group is responsible for implementing physical activity and healthy nutrition-promoting activities	Children aged 7–12 years	3 years	Principals, working group chairs, members of the steering committee	The mutual adaptation between top-down and bottom-up influences were key elements of the intervention. Feedback loops and the health promotion advisors played a crucial role in navigating between influences

### Quality assessment

A quality assessment was made on all included studies by two authors independently (LF and CD). The Mixed-Methods Appraisal Tool Version 2018 (MMAT) was applied due to its ability to appraise methodological quality from a range of designs, including qualitative research, quantitative descriptive research and mixed-methods studies^(20)^. MMAT includes two screening questions, followed by five questions per study design, where responses can either be ‘yes’, ‘no’ or ‘can’t tell’. Questions explored the following across the respective study designs: appropriateness of the chosen study design and methods, interpretation and translation of findings, potential risks of bias or inconsistencies in results. It is discouraged to calculate an overall score from the ratings of each criterion^(20)^; therefore, the ratings were considered individually. Any discrepancies in scoring were resolved through discussion until consensus was reached. The detailed assessment can be found in online Supplementary Material 2.

### Data synthesis

Enablers and barriers that were reported as influential to sustained implementation were deductively coded based on the ISF, developed by Shelton *et al*.^(11)^ The ISF was chosen due to its ability to capture multi-level factors that affect longer-term sustainability of interventions. The framework identifies twenty-one dynamic factors across its five domains: ‘outer contextual factors’, ‘inner contextual factors’, ‘processes’, ‘characteristics of the interventionists’ and ‘characteristics of the intervention’, which, when applied, highlight salient factors for consideration^(11,15)^.

**Fig. 1:**
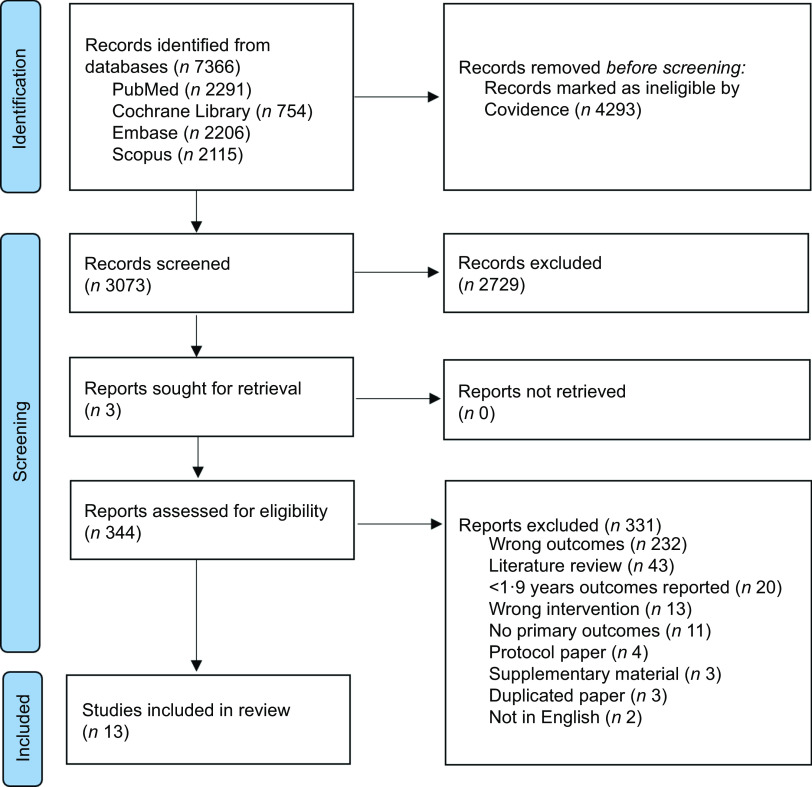
Preferred Reporting Items for Systematic Review and Meta-Analyses flow diagram

Coding was performed by two authors who were experienced with qualitative research (LF and CD), using the coding manual and definitions developed by Shoesmith *et al*.^(15)^ All qualitative and descriptive quantitative factors from included studies were coded aligned with the twenty-one factors that sit within the five domains of the ISF (see online Supplementary Material 4). Any discrepancies in coding were resolved by consensus or by a third author (HT). Enablers and barriers to sustained implementation were categorised under all domains of the ISF, including frequency counts of the number of programmes which identified those factors (including the number of corresponding articles that identified the factors). See Table 3 and Fig. 1 for results displayed across the framework domains.

**Table 3:** Number of programmes which identified barriers and enablers to implementation and sustainability according to the Integrated Sustainability Framework domains and factors

Integrated Sustainability Framework domains and factors	Number of programmes which identified barriers to implementation and sustainability (*n* 13 articles identified barriers)	Number of programmes which identified enablers to implementation and sustainability (*n* 13 articles identified enablers)
Outer contextual factors	(*n* 8 articles)	(*n* 13 articles)
Socio-political context	1^(27)^	4^(25,27,29–31)^
Funding environment and availability	3^(23,25,29,33)^	6^(23,25–29,31,33)^
External partnerships and leadership/environmental support	4^(24,27,30,34)^	9^(22,24,25,27,29–34)^
Values, needs and priorities	4^(22,25,29,30,33)^	3^(30,33,34)^
Inner contextual factors	(*n* 13 articles)	(*n* 9 articles)
Programme champions	0	3^(25,27,29,33)^
Organisational leadership/support	6^(24–30,32)^	7^(23–25,27,29,30,33,34)^
Organisational readiness/resources	11^(22–34)^	5^(24,25,27,29)^
Organisational stability	4 ^(23–25,27,29)^	2 ^(23,27,30,32)^
Processes	(*n* 11 articles)	(*n* 11 articles)
Partnership/engagement	3 ^(25,26,28–30)^	4 ^(22,25,29,30,33)^
Training/supervision/support	5 ^(25,26,28,29,32–34)^	7 ^(22,24,25,29,30,32–34)^
Programme evaluation/data	4 ^(25,27,29,31,34)^	1 ^(27)^
Adaptation	0	0
Communications and strategic planning	5 ^(24,25,27,29,31,32)^	6 ^(24–30,34)^
Characteristics of the interventionists and population	(*n* 5 articles)	(*n* 5 articles)
Implementer characteristics	2 ^(24,33)^	2 ^(24,25,29)^
Implementer benefits and stressors	0	1 ^(23)^
Implementer skills/expertise	1 ^(25,29)^	2 ^(25,29,32)^
Population characteristics	2 ^(23,25,29)^	0
Characteristics of the intervention	(*n* 9 articles)	(*n* 11 articles)
Adaptability of EBI/fidelity	0	4 ^(25,27,29,31,34)^
Fit with context/population/organisation	7 ^(22–26,28–31)^	7 ^(22,23,25,26,28–32)^
Perceived benefits	0	6 ^(23–26,28,29,31)^
Perceived need	0	1 ^(25,29)^

## Results

### Study selection

**Fig. 2:**
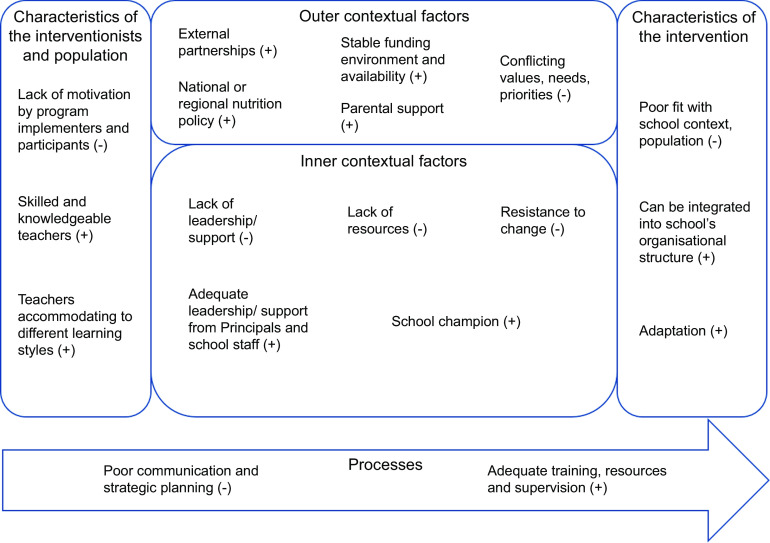
Summary of findings categorised into the Integrated Sustainability Framework domains. Enabling factors are depicted with a (+) and barriers are depicted with a (–)

Identification and selection of studies are summarised in Fig. 2. The search strategy yielded 7366 studies and 4293 duplicates was removed, leaving 3073 articles. Of this, 2729 studies were excluded based on the title and abstract. A total of 331 full texts were excluded primarily due to the wrong outcomes being reported. A total of thirteen studies met the eligibility criteria.

### Quality assessment

Refer to online Supplementary Material 2 for the full quality assessment. All studies received a ‘yes’ for the first two screening questions which asked whether studies had a clear research question and had data collected which would allow the research question to be addressed. Four of five studies with a mixed-methods design did not adequately provide a rationale for utilising a mixed-methods design or have adequate integration of their mixed methods, therefore receiving a ‘no’ or ‘can’t tell’, in one or more criteria (5·1, 5·2, 5·3)^(22–25)^. The qualitative studies were of higher quality and only two studies had inadequate use of quotes to substantiate themes, resulting in ‘can’t tell’ across 1·3, 1·4 and 1·5 of the criteria^(26,27)^. The quantitative descriptive study received mostly ‘yes’ for the criterion; however, it was unclear whether the survey that was utilised in the study had been pre-tested, thus receiving ‘can’t tell’ in response to ‘Are the measurements appropriate?’^(28)^. Although there were some inconsistencies across methodological quality, the authors felt that they were not sufficiently substantial to impact the overall integrity of the study.

### Study characteristics

The thirteen included studies which reported on eleven programmes (average of 4·6 years and a range of 8 years in duration) were from eight countries: Canada^(25,29,30)^, Australia^(26,28)^, Ireland^(31)^, England^(32)^, USA^(23,24,33)^, the Netherlands^(27)^, Thailand^(34)^ and New Zealand^(22)^. Only five studies^(22,28–31)^ reported on interventions lasting ≥ 5 years in duration. Key characteristics of individual studies are reported in Tables 1 and 2. The majority of the programmes^(22–25,27,29,30,32–34)^ reported on interventions that were multi-component by design or undertook a whole-school approach to either improve the school food environment or health-promoting culture, whereas three studies^(26,28,31)^ only implemented specific dietary interventions for the classroom and home environments.

All programmes reported similar aims of promoting health through improving diet, some included a physical activity component and all had the long-term objective of reducing risk factors for chronic diseases or obesity. Seven studies utilised qualitative design via individual interviews or focus groups^(26,27,30–34)^, five studies^(22–25,29)^ applied mixed-methods designs and one study^(28)^ used a quantitative (descriptive) design. All studies sought to understand the enablers and barriers of programme sustainment via the perspectives of programme implementers such as classroom teachers, physical education teachers and school champions^(22–25,27,29–34)^. Additional stakeholder perspectives included that of school principals, administration staff, programme supporters and health promotion officers.

### Review outcomes

Studies were categorised by programme titles in online Supplementary Material 3. The following eleven programmes were evaluated: Action Schools! BC (AS! BC); Crunch&Sip; Food Dudes; Food for Fitness; Health Promoting Schools (HPS); Healthy Choices; Kansen in Eindhoven voor GezinsAAnpak met Fontys which translates to ‘Chances in Eindhoven for a family-based approach by Fontys’ (KEIGAAF); New Moves; Pathways; the Diamond Level Health-Promoting Schools (DLHPS); and Wellbeing and Vitality in Education (WAVE). Deductive coding of the study results revealed thirty-four factors that influenced the sustainable implementation of programmes. The codes were further synthesised into five overarching domains which guided the formulation of recommendations, a summary of which can be seen in Fig. 1.

### Barriers to programme implementation and sustainability

Fifteen barriers were identified to impede on programme implementation and sustainability across all domains of the ISF (Table 3). The most frequently identified outer contextual factors were ‘Values, needs and priorities’ (*n* 4 programmes) and ‘External partnerships and leadership/environmental support’ (*n* 4). For instance, the Healthy Choices^(33)^ programme reported that there were time constraints due to state-mandated testing (to gather student data on performance across school curricula) which took priority. For the inner context, ‘Organisational leadership/support’ (*n* 6) and ‘Organisational readiness/resources’ (*n* 11) were most frequently reported as barriers. For example, Crunch&Sip reported a lack of clarity and overlap of roles undertaken by nongovernmental organisations and Local Health District staff which increased inconsistent delivery and decreased programme efficiency^(26)^. ‘Communications and strategic planning’ (*n* 5) and ‘Training/supervision/support’ (*n* 5) were identified as the most common barrier for processes. Food for Fitness reported that inefficient planning processes and poor class organisation were barriers to effective management of the programme^(32)^. For the characteristics of the interventionists and population, few barriers were reported; however, ‘Implementer characteristics’ (*n* 2) and ‘Population characteristics’ (*n* 2) were important to consider. Lack of motivation and reluctance to change were identified by Pathways and Healthy Choices, respectively^(24,33)^. In terms of characteristics of the intervention, ‘Fit with context/population/organisation’ (*n* 7) was the only barrier that was frequently perceived. It was identified that educational and cultural priorities limited health promotion and sustainability for Health-Promoting Schools in Nova Scotia, Canada^(30)^.

### Enablers to programme implementation and sustainability

Nineteen enablers were identified that helped support programme implementation and long-term sustainability across all domains of the ISF (Table 3). The most frequently identified outer contextual factors were ‘Funding environment and availability’ (*n* 6 programmes) and ‘External partnerships and leadership/environmental support’ (*n* 9). For WAVE in New Zealand, it was identified that cultural linkages with local Indigenous groups were essential for intersectoral collaboration between the health and education sectors^(22)^. For the inner context, ‘Organisational leadership/support’ (*n* 7) was identified as a significant enabler to programme sustainability. For example, the support of school staff, the principal and parents was integral for KEIGAAF^(27)^. School staff facilitated the integration of activities and policies within the school and schools that were most active in implementation had a principal who supported the working groups. ‘Training/supervision/support’ (*n* 7) was the most common enabler for processes involved in programme implementation. Action Schools! BC considered support from the central team, having access to resources and adequate training with follow-up support to be enablers to programme implementation^(25,29)^. Very few factors were identified for characteristics of the interventionists and population; however, ‘Implementer skills/expertise’ (*n* 2) and ‘Implementer characteristics’ (*n* 2) were frequently noted. For example, stakeholders of Food for Fitness identified that using skilled and knowledgeable staff with a practical and applied approach, in addition to being able to recognise the multiple learning styles involved in the delivery of lessons, was beneficial^(32)^. For the characteristics of the intervention, ‘Fit with context/population/organisation’ (*n* 7) was more common as an enabling consideration. A significant enabler that affected the sustainability of Food Dudes was whether the programme was embedded in an organisational structure that offered support through pre-existing healthy eating policies, which reflected the ethos and commitment of the school^(31)^.

## Discussion

The aim of this systematic literature review was to explore the enablers and barriers that influence the sustained implementation of school-based nutrition programmes for children aged 5–14 years. Various enablers and barriers were identified which influenced the sustainable implementation of eleven international programmes, which were synthesised across all domains of the ISF. Barriers were more frequently noted in relation to ‘inner contextual factors’, whereas enablers were more prevalent in ‘outer contextual factors’. The findings suggest that careful attention should be directed towards understanding the factors which influence the sustainability of effective and efficacious programmes, to improve the integration of the programme itself into government systems.

### Outer contextual factors

In terms of outer contextual factors, the socio-political context, funding environment and external partnerships and values/priorities were all influential enablers to long-term programme sustainability and were deemed the most influential to programme sustainment (*n* 13 studies). Shoesmith *et al*.^(15)^ support this finding, highlighting that the aforementioned factors are important enablers to programme sustainment; however, their review reported on ‘inner contextual factors’ being most influential to intervention sustainment, which differs to the finding of our review. Secure and long-term funding from provincial or national levels of government, even if it involved a budget reduction from the roll-out phase, was crucial to sustainability^(25,26,29,31)^. This finding was triangulated and supported by reviews by Stirman *et al*.^(13)^ and Shoesmith *et al*.^(15)^, which reported on funding, being a key factor that influences programme sustainment. Ultimately, what enabled programmes that had been implemented for at least 5 years to continue was the funding within a supportive socio-political context. Lasting partnerships and strong relationships across government, which may evolve alongside policy changes, were an essential strategic component that underpinned funding sustainment.

It is inevitable that health promotion in school settings is impacted by political ideology and stability in government policy for health promotion activities. High level policy and institutional anchoring, pressure from national health-promoting trends and adopting provincial or local guidelines enabled the continuation of Health-Promoting Schools^(35)^. Hoelscher *et al*.^(36)^ acknowledged the importance of considering socio-environmental factors, such as unhelpful pre-existing policies and the influences of the food and beverage industries. The review by Shoesmith *et al*.^(15)^ recognises that external socio-political landscape is essential in supporting programme sustainment through policies, mandates, regulations and provision of on-going financial support. The Academy of Nutrition and Dietetics, the leading nutrition association in the USA and considered a trusted, reputable voice for nutrition-related issues, has recommended policy and environmental interventions as feasible and sustainable ways to support healthful lifestyles and reduce childhood obesity^(36)^.

#### Implications

It is crucial, therefore, that cross-department governance and collaborations are strengthened to plan for long-term funding and to establish a model that plans for the sustainment of programmes from their initiation. It has been recommended to implement programmes into the school curriculum and within schools with pre-existing health policies, as these have been identified as enablers to long-term adoption of programmes^(37,38)^. Future research should investigate what factors enable long-term funding, as current studies revealed that funding insecurity was a significant contributor to programme discontinuation^(39,40)^.

### Inner contextual factors

Adequate organisational leadership/support^(23–25,27,29,30,33,34)^, readiness/resources^(23–25,27,29,32)^ and programme champions^(25,27,29,33)^ were considered as the most important enablers for programme implementation within the ‘inner context’, which is supported by previous reviews^(12,13,15)^. Insufficient support, unclear communication and inadequate role clarification negatively influenced the efficiency of programme implementation, this was due to uncertainty around role requirements leading to unintentional overlap of tasks^(26)^. Franks *et al*.^(41)^ and Rogers *et al*.^(42)^ demonstrated that successful programme dissemination and implementation require enthusiasm, commitment and collaboration between key stakeholders involved. The support and involvement of a school principal and other administration staff were deemed crucial for the successful implementation of the Coordinated Approach to Child Health programme, in addition to the identification of required resources which benefited Planet Health^(41)^. Having the commitment of school leadership enables the integration of programme components into organisational processes. These are all factors which influence an organisation’s climate and readiness for sustained implementation of a nutrition intervention^(39)^.

#### Implications

Existing and future programmes should prioritise involving various school staff and members of administration to increase support networks and resources for programme implementation^(41)^. Stakeholders and programme implementers should have clear definitions of expectations and roles and be empowered to work in a collaborative manner^(42)^. Upcoming research should further investigate the procedures which encourage a positive organisational climate and ongoing staff support, as these factors help to increase organisational capacity to take ownership of the programme and to have a successful and sustainable programme^(39)^.

### Processes

Partnership/engagement, training/supervision/support, programme evaluation, communications and strategic planning were important factors which influenced sustainable programme implementation^(22,24–34)^. Meeting staff needs for professional development and curriculum support was deemed integral; however, the messaging during training had to be clear and practice orientated^(22,27,30)^. Teachers and programme implementers found having access to specialist health promotion expertise and follow-ups with programme coordinators to be beneficial^(22,25,29)^. Due to time constraints, recording evaluation data was considered disruptive by teachers^(31)^. These findings are supported by other long-term health-promoting programmes where effective and on-going training of multidisciplinary teams (such as classroom and physical education teachers and food service staff) ensured the long-term delivery of programme curricula^(41)^. Teachers were more willing to be enthusiastic when prepared lessons that were aligned with education standards were provided with adequate training and flexibility for the delivery of the material^(41)^. Shelton *et al*.^(15)^ and Herlitz *et al.* ^(12)^ have identified that training/supervision/support is a significant process factor which can either provide opportunities for upskilling, whereas a lack thereof is a barrier to sustainability.

#### Implications

Health-promoting programme developers should ensure adequate training and supervision for programme implementers to allow for capacity building, empowerment and a clear vision of programme goals. Due to the pressures placed upon teachers and administrative staff, such as managing an already crowded curriculum and the inevitable time restraints for extracurricular activities, it is recommended that programmes engage or embed programme coordinators, who are familiar with the education system. A programme coordinator can support teachers with practical ways to integrate learning about food and nutrition within the existing curriculum and to provide monitoring of implementation, as well as identify sources of resource provision.

### Characteristics of interventionists and population

Implementer and population characteristics, in addition to implementer skills/expertise and benefits, were factors that were considered to also influence long-term programme implementation^(23–25,29,32,33)^. Having committed teachers was seen as very important to involve children, parents and administration staff^(24)^. It was favourable when teachers were skilled, knowledgeable and used a practical and applied approach, which included recognising the multiple learning styles of children^(32)^, factors which were emphasised by a 2020 systematic review by Herlitz *et al*.^(12)^ The findings are also supported by Cassar *et al*.^(43)^ which recognised that optimal characteristics of teachers included: high self-efficacy, flexibility towards adaptations and changes in practice and policy, and strong motivation. Teachers were more likely to continue implementing a programme if they observed enthusiasm from the children and believed in the advantage of the programme to students^(43)^.

#### Implications

Existing and future programme developers should learn and understand the factors which increase teacher self-efficacy, confidence and intrinsic motivation to sustain a health-promoting programme. Training and professional development opportunities can be used to ensure that the appropriate skills are developed that will enable an implementer to confidently deliver the programme.

### Intervention characteristics

A programme’s lack of fit with the school’s context, population and organisation was a barrier to long-term programme implementation^(22–26,28–31)^. Conversely, when a programme was adaptable and well-aligned with a school’s context, it was an enabling factor^(22,23,25,26,28–32)^. When the programme had perceived benefits and needs, this helped to facilitate implementation^(23–26,28,29,31)^. These findings are supported by the Association for Supervision and Curriculum Development (ASCD), an international non-for-profit organisation that advocates for policies and practices which enhance a child’s education and access to equity. ASCD recommends that health programmes need to understand the cultural anchors of schools and need to be integrated within the core mandates, constraints, processes and preoccupations of education systems, leading to an integration of health across whole of government in order to achieve sustainability^(44)^. Similarly, Rogers *et al*.^(42)^ identified that integrating interventions into existing curricula optimised perceived relevance by school stakeholders, resulting in 90 % of teachers positively responding to the programme design. Integrating a whole school approach, via an adjustment to the school ethos and culture, was also shown to elicit a positive school environment and assist sustainable implementation of health-based programmes^(13,42,45,46)^.

#### Implications

Stakeholders and programme implementers should acknowledge and seek to understand the unique features, cultural anchors and priorities of the schools that will implement health-based programmes^(44)^. It is imperative for health-promoting programmes to integrate with educational values to ultimately enable strong partnerships across education and health sectors. These acknowledgements should lead to appropriate adaptations to implementation processes and programme components to best suit the school context, to ensure its longevity and resilience^(47)^.

### Future directions

Based on this review, the following strategies should be considered to support the sustainment of food and nutrition-based programmes in the school setting and to address key barriers: (i) programme implementers should establish and foster robust relationships with local institutions, businesses and stakeholders who can support or advocate for essential resources; (ii) governance structures should align with political and local environmental enablers and seek to establish a long-term funding model which may be different to the initiation funding phase and (iii) programmes should be designed to be flexible to accommodate to the unique needs of schools within diverse societal contexts. We further recommend that future research investigates the relative weighting of sustainability determinants to establish which are the critical components for focusing strategies on.

### Strengths and limitations

A limitation regarding the evidence obtained was the language bias towards only including papers that were published in the English language. Therefore, the findings may not be transferable to all countries and cultures since the included papers primarily had Western-centric perspectives. A further limitation to the evidence was that all included studies were located in high-income countries. This limits the ability for global scale implications to be drawn. Another element which was not captured in the review was the nature and extent to which programme characteristics potentially were adapted locally over time.

A methodological strength was that the deductive coding was based off a sustainability specific framework which acknowledges the dynamic interplay between schools and their inner and external climates. This review provides the most up-to-date overview of what contributes to the sustainability of international school-based food-nutrition interventions and reports on the relatively small number of programmes that survive past 2 years. A greater understanding of what can be built into programmes from their outset to make them more robust and adaptable to economic, political and environmental changes, is crucial to ensure that school-based programmes are sustainable long-term.

## Conclusion

The ISF may be useful in a feed forward approach to programme planning, to ensure that elements of the inner and outer environments are taken into consideration to plan for programme longevity. This review presents key features of school-based nutrition programmes that enable and interfere with long-term (≥ 2 years) implementation. The findings can be used as guidelines to plan for sustainable outcomes in primary school settings and to ensure that funding attributed to school-based approaches is money well spent.

## Supplementary material

For supplementary material accompanying this paper visit https://doi.org/10.1017/S1368980023001647. click here to view supplementary material
