# Applying the Integrated Sustainability Framework to explore the long-term sustainability of nutrition education programs in schools: A systematic review – CORRIGENDUM

**DOI:** 10.1017/S1368980023002483

**Published:** 2023-12

**Authors:** Leila Isabella Fathi, Jacqueline Walker, Clare Frances Dix, Jessica Rose Cartwright, Suné Joubert, Kerri Amelia Carmichael, Yu-Shan Huang, Robyn Littlewood, Helen Truby

In the published article, ‘Shelton, RC, Cooper, BR & Stirman, SW (2018) The sustainability of evidence-based interventions and practices in public health and health care. *Ann Rev Public Health* **39**, 55–76. doi: 10.1146/annurev-publhealth-040617-014731’ was incorrectly listed as reference 11, when this should have been ‘Moore, J.E., Mascarenhas, A., Bain, J. *et al*. Developing a comprehensive definition of sustainability. *Implementation Sci* **12**, 110 (2017).’

The article has now been updated to amend this error.
