# Supplementary material for: Applying the Integrated Sustainability Framework to explore the long-term sustainability of nutrition education programmes in schools: a systematic review
Source: Public Health Nutr. 2023 Aug 7;26(10):2165–79. doi: 10.1017/S1368980023001647 (PMC10564612; doi:10.1017/S1368980023001647)
Supplement: Supplementary file 1 [file S1368980023001647sup.zip › S1368980023001647sup001.docx]

## Supplementary Material 1: Academic Database Search Table

| **Database** | **Detailed Search Terms/Strategy** | **Number of Results Obtained** |
| --- | --- | --- |
| Cochrane Library | ((((([mh ^Students] OR [mh ^Child] OR [mh Adolescent] OR student*:ti,ab OR child:ti,ab OR children:ti,ab OR adolescent*:ti,ab) AND (([mh "Health Promotion"]) OR (program*:ti,ab OR promotion*:ti,ab OR intervention*:ti,ab))) AND ([mh ^Schools] OR "school-based":ti,ab OR ("elementary" NEAR/2 school*):ti,ab OR ("middle" NEAR/2 school*):ti,ab OR ("primary" NEAR/2 school*):ti,ab OR ("high" NEAR/2 school*):ti,ab OR ("secondary" NEAR/2 school*):ti,ab)) AND ([mh Dietetics] OR [mh Diet] OR diet*:ti,ab OR nutrition*:ti,ab OR "healthy eating":ti,ab OR fruit:ti,ab OR vegetable*:ti,ab)) AND ([mh "Program Evaluation"] OR engagement:ti,ab OR barrier*:ti,ab OR facilitat*:ti,ab OR enabler*:ti,ab OR "long-term":ti,ab OR sustain*:ti,ab OR evaluat*:ti,ab)) NOT ([mh adult] NOT [mh child]) | 754 |
| Embase | (((((Students/ OR Child/ OR exp Adolescent/ OR student*.ti,ab. OR child.ti,ab. OR children.ti,ab. OR adolescent*.ti,ab.) AND ((exp Health Promotion/) OR (program*.ti,ab. OR promotion*.ti,ab. OR intervention*.ti,ab.))) AND (Schools/ OR school-based.ti,ab. OR elementary school*.ti,ab. OR middle school*.ti,ab. OR primary school*.ti,ab. OR high school*.ti,ab. OR secondary school*.ti,ab.)) AND (exp Dietetics/ OR exp Diet/ OR diet*.ti,ab. OR nutrition*.ti,ab. OR healthy eating.ti,ab. OR fruit.ti,ab. OR vegetable*.ti,ab.)) AND (exp Program Evaluation/ OR engagement.ti,ab. OR barrier*.ti,ab. OR facilitat*.ti,ab. OR enabler*.ti,ab. OR long-term.ti,ab. OR sustain*.ti,ab. OR evaluat*.ti,ab.)) NOT (exp adult/ NOT exp child/) | 2206 |
| PubMed | ((((("Students"[Mesh:NoExp] OR "Child"[Mesh:NoExp] OR "Adolescent"[Mesh] OR student*[Title/Abstract] OR child[Title/Abstract] OR children[Title/Abstract] OR adolescent*[Title/Abstract]) AND (("Health Promotion"[Mesh]) OR (program*[Title/Abstract] OR promotion*[Title/Abstract] OR intervention*[Title/Abstract]))) AND ("Schools"[Mesh:NoExp] OR "school-based"[Title/Abstract] OR "elementary school*"[Title/Abstract] OR "middle school*"[Title/Abstract] OR "primary school*"[Title/Abstract] OR "high school*"[Title/Abstract] OR "secondary school*"[Title/Abstract])) AND ("Dietetics"[Mesh] OR "Diet"[Mesh] OR diet*[Title/Abstract] OR nutrition*[Title/Abstract] OR "healthy eating"[Title/Abstract] OR fruit[Title/Abstract] OR vegetable*[Title/Abstract])) AND ("Program Evaluation"[Mesh] OR engagement[Title/Abstract] OR barrier*[Title/Abstract] OR facilitat*[Title/Abstract] OR enabler*[Title/Abstract] OR "long-term"[Title/Abstract] OR sustain*[Title/Abstract] OR evaluat*[Title/Abstract])) NOT (adult[mh] NOT child[mh]) | 2291 |
| Scopus | (((INDEXTERMS(Students OR Child OR Adolescent)) OR (TITLE-ABS(student* OR child OR children OR adolescent*))) AND ((INDEXTERMS("health promotion")) OR (TITLE-ABS(program* OR promotion* OR intervention*))) AND (INDEXTERMS("primary school") OR (TITLE-ABS("school-based" OR "elementary school*" OR "middle school*" OR "primary school*" OR "high school*" OR "secondary school*"))) AND ((INDEXTERMS(Dietetics OR Diet)) OR (TITLE-ABS(diet* OR nutrition* OR "healthy eating" OR fruit OR vegetable* OR "fresh produce"))) AND ((INDEXTERMS("Program Evaluation")) OR (TITLE-ABS(engagement OR barrier* OR facilitat* OR enabler* OR "long-term" OR sustain* OR evaluat*)))) AND NOT ((INDEXTERMS(adult)) AND NOT (INDEXTERMS(child))) | 2115 |
