# Supplementary material for: Applying the Integrated Sustainability Framework to explore the long-term sustainability of nutrition education programmes in schools: a systematic review
Source: Public Health Nutr. 2023 Aug 7;26(10):2165–79. doi: 10.1017/S1368980023001647 (PMC10564612; doi:10.1017/S1368980023001647)
Supplement: Supplementary file 1 [file S1368980023001647sup.zip › S1368980023001647sup002.docx]

### Supplementary Material 2: Quality Assessment of Included Studies

| **Citation** | **Publication Date** | **Study Design** | **S1** | **S2** | **1.1** | **1.2** | **1.3** | **1.4** | **1.5** | **4.1** | **4.2** | **4.3** | **4.4** | **4.5** | **5.1** | **5.2** | **5.3** | **5.4** | **5.5** |
| --- | --- | --- | --- | --- | --- | --- | --- | --- | --- | --- | --- | --- | --- | --- | --- | --- | --- | --- | --- |
| Biggs J., et al. | 2014 | Qualitative | Y | Y | Y | Y | Y | U | U |  |  |  |  |  |  |  |  |  |  |
| Calder K., et al. | 2017 | Mixed methods | Y | Y |  |  |  |  |  |  |  |  |  |  | Y | U | Y | Y | Y |
| Friend S., et al. | 2014 | Mixed methods | Y | Y |  |  |  |  |  |  |  |  |  |  | Y | U | Y | Y | Y |
| Gittelsohn J., et al. | 2003 | Mixed methods | Y | Y |  |  |  |  |  |  |  |  |  |  | U | U | U | Y | Y |
| Greaney M., et al. | 2014 | Qualitative | Y | Y | Y | Y | Y | Y | Y |  |  |  |  |  |  |  |  |  |  |
| Hayes C., et al. | 2019 | Qualitative | Y | Y | Y | Y | Y | Y | Y |  |  |  |  |  |  |  |  |  |  |
| McIsaac J., et al. | 2015 | Qualitative | Y | Y | Y | Y | Y | Y | Y |  |  |  |  |  |  |  |  |  |  |
| Middleton G., et al. | 2012 | Qualitative | Y | Y | Y | Y | Y | Y | Y |  |  |  |  |  |  |  |  |  |  |
| Nathan N., et al. | 2017 | Quantitative (descriptive) | Y | Y |  |  |  |  |  | Y | Y | U | Y | Y |  |  |  |  |  |
| Naylor P., et al. | 2010 | Mixed methods | Y | Y |  |  |  |  |  |  |  |  |  |  | N | N | Y | Y | Y |
| Naylor P., et al. | 2015 | Mixed methods | Y | Y |  |  |  |  |  |  |  |  |  |  | Y | Y | Y | Y | Y |
| Phaitrakoon J., et al. | 2014 | Qualitative | Y | Y | Y | Y | Y | Y | Y |  |  |  |  |  |  |  |  |  |  |
| Verjans-Janssen S., et al. | 2020 | Qualitative | Y | Y | Y | Y | U | U | U |  |  |  |  |  |  |  |  |  |  |
