# Supplementary material for: Applying the Integrated Sustainability Framework to explore the long-term sustainability of nutrition education programmes in schools: a systematic review
Source: Public Health Nutr. 2023 Aug 7;26(10):2165–79. doi: 10.1017/S1368980023001647 (PMC10564612; doi:10.1017/S1368980023001647)
Supplement: Supplementary file 1 [file S1368980023001647sup.zip › S1368980023001647sup003.docx]

### Supplementary Material 3: Data extraction of enablers and barriers to sustainability and implementation of included programs

| **Program** | **Enablers - sustainability** | **Enablers - implementation** | **Barriers - sustainability** | **Barriers - implementation** |
| --- | --- | --- | --- | --- |
| Action Schools! BC (23, 24) | NR | - Having a school 'leader/champion' - Support from the AS! BC central team - Access to resources - Access to funding - Community and school engagement  - Official integration of the AS! BC HE activities within the curriculum - Keeping the HE model relevant, fresh and responsive to teachers' needs - Organisational climate - Adopting and implementing AS! BC HE and the Provincial Guidelines for Food and Beverage Sales in the Schools - Top priority - Schools in the least urbanised areas - Teachers with high self-efficacy - Teachers who had healthy eating training - Training and support  - It's easy to use - School commitment - Community need | NR | - Lack of time as most common barrier - Lack of resources - Lack of leadership caused by high staff turnover - Lack of momentum  - Cost not being reasonable - Larger school - Use was lower in schools where the principals strongly agreed or agreed that the climate was supportive of HE - Lack of school resources - Lack of time - Lack of knowledge of healthy living - Student reaction - Evaluation (logging) - Inconsistent messages in the community - High staff turnover - Admin support - Weather |
| Crunch&Sip (20, 22) | NR | **-** Principals agreed that: there is evidence that the program increases student consumption of vegetables and fruit  - Their school had sufficient resources to implement the program (e.g. money, time)  - The program needed to be simple to appeal to teaching staff | NR | - That the program would be too difficult to implement  - Principals who agreed that implementing the program is far less important than other priorities within the school  - Schools identified the two-step delivery model (registration and certification) as a major barrier to implementation. Teachers found the process "confusing".  **-** Minimal follow-up after certification had been received - Lack of clarity and overlap of roles undertaken by NGO and LHS staff increased inconsistent delivery and decreased program efficiency |
| Food Dudes (26) | - Having secured funding. Budgetary reduction.  - Having the program embedded in an organisational structure which offered support to the program e.g. pre-existing healthy eating policies, which reflected the ethos and commitment of the school. - The presence of pre-existing whole school policies was a key factor. - Long term sustainability was aided by a variety of pre-existing healthy eating measures and parental support. | - Fidelity to core components - Incentives and rewards for the children - Adaptations to FD were seen as enabling factors in ensuring successful implementation e.g. adaptations to program delivery were made for children with special needs and/or serious aversions to specific fruit or vegetables and the timing of the intervention was customised to suit the school timetable.  - Simplicity of intervention | - If the program is not embedded in an organisational structure which offered support to the program e.g. pre-existing healthy eating policies, which reflected the ethos and commitment of the school. - Insufficient communication between stakeholders at all levels. | - Time required to implement and record uptake of the program - Lack of canteens to provided logistical challenges. - Disruptions by researchers |
| Food for Fitness (27) | NR | - Using skilled and knowledgeable staff with a practical and applied approach. - Recognition of the multiple learning styles involved in the delivery of lessons. - Program should be acceptable for the school environment. - Clear consistent messages in training and advice. | - Lack of extended school support. | - Limited staff and facilities as a restrictive capacity issue.  - Poor class organisation and timing.  - Inefficient planning processes. |
| Health Promoting Schools (HPS) (25) | - High level policy and institutional anchoring of health-promoting schools (HPS). - Professional developing and learning. - Preparing and planning for school development. - Leadership and management practices. - Relational and organisational context. - School partnerships and networking. - Student participation and engagement | - Supportive leadership from the principal who also provided flexibility to enable support among school staff. - Distributed leadership through shared responsibilities for activity implementation.  - Support provided by community partners and committed school staff.  - Student-centred learning, an emphasis on skill development and fostering enjoyment among students.  - Culture for health promotion. Integration of health into priorities and the culture of the school. HPS philosophy shared by their respective school districts.  - Support from entire school staff to support implementation of school-wide activities. - Having a committee or team that meets to plan activities.  - Collaborative engagement. | - Educational and cultural priorities limiting health promotion and sustainability. | - Limited organisational capacity. - Limited engagement in community partnerships. - Challenges of capacity and time. - Insufficient staff and volunteer capacity. - Health promotion not being a school priority.  - Limited dedicated resources.  - Conflicting policy, priorities and resources and finding it challenging to balance health promotion initiatives with current academic priorities. |
| Healthy Choices (30) | - Reducing MCAS (state-mandated testing) related pressures - Continued buy-in and support from administration and staff (maintaining existing supports while acquiring additional ones) - Necessity of parental/community support - Importance of future funding - Maintaining contact with outside experts | - Having a program champion and employing a team approach. - School coordinator's strong relationships with faculty and staff. - Administrative and staff buy-in - External support offered (e.g. regional coordinators, program trainings. | NR | - Time constraints owing to state-mandated testing - Unwillingness of some teachers to use Planet Health - Difficulty in training teachers in Planet Health - Perceived reluctance of food service personnel to make changes - Budget limitations make it difficult to make changes in dining services. - General time constraints. |
| KEIGAAF (21) | - The intervention being characterised by a high degree of adaptation and local tailoring. | - Practical support of health promoting advisors. Some working groups required guidance and encouragement of the HP advisor. The HP advisor sought the best strategy to guide and encourage a working group. A good match between these strategies and the needs of the working group facilitated implementation.  - Financial support - Feedback loops in the intervention supported implementation. - National health promoting trends. The guidelines of the national HPS committee concerning the HPS certificates enabled all schools to set priorities. Other national trends, such as the EU school fruit program or national initiatives such as National Sports Week, also facilitated the schools in the implementation of PA and healthy eating promotion.  - Top-down influence from school board. The school board's demand to obtain the HPS certificates supported the KEIGAAF approach by accelerating the implementation of PA and nutrition-promoting activities.  - A good starting situation school health promotion- the working group considered that the current situation had to change (limited PA or healthy nutrition promotion at the school). - Support of school staff, principal, and parents. School staff support facilitated the integration of activities and policies within the school. Schools that were most active in implementation had a principal who supported the working group, agreed on decisions made by the working group, demanded that the rest of the school staff support the implementation of this plan.  - Employee turnover. In most schools, the employee turnover facilitated the implementation of the intervention, because the new members were more practice, had more decisional power, or because the changes could be more easily implemented given the teachers' unfamiliarity with the old practices. - Champion. A factor facilitating the integration of the activities within a school was the presence of a champion. - Positive dynamics. Good interaction between working group members, including constructive communication between the working group members and the HP advisor, facilitated the process of developing and implementing. - Practice-orientated thinking of the working groups facilitated implementation - Adaptation. The KEIGAAF intervention was characterised by a high degree of adaptation and local tailoring which facilitated the implementation of activities that were suitable for the local context. It also enhanced feelings of ownership and sustainability of the bottom-up approach. | - Practice orientated thinking of the working groups did not facilitate the development of a deliberated and sustainable plan. | - Feedback loops. The results of the behavioural measurements of the children were demotivating for some schools because, in the short term, the children did not improve in their behavioural outcomes.  - Nutrition as intervention topic was a barrier to implementation due to lack of support from multiple actors.  - Lack of potential partners. A lack of potential partners or limited collaborations between school and potential partners hindered the implementation of comprehensive PA and healthy nutrition promotion.  - Starting situation school health promotion. When the working group considered that their school was already making much effort regarding PA and nutrition promotion, implementation was hindered.  - Low parental support and involvement with school activities (whether health-related or not). - At some schools, high employee turnover inhibited implementation because of poor communication between the leaving employee(s) and the new employee(s) or uncertainty about division of tasks.  - Misinterpretation of the intervention approach and the intervention objectives inhibited implementation. |
| New Moves (28) | - Teachers believing in the importance of the program. - Feelings of support for the class by school administrators. - Receiving teacher training on the New Mooves program from the research team. | NR | - Low motivation among girls. - Class size (too large or too small). Large class sizes led to difficulties in class management, whereas class sizes that were too small were not sustainable due to school policies for minimal class sizes.  - Lack of adequate time. - Lack of adequate finances. - Teacher turnover. | NR |
| Pathways (29) | NR | - Having committed teachers was seen as very important to the overall involvement of parents, children and administration. - Teachers who worked together and supported one another. - Positive school climate - Good communication and support when the administration and teachers worked together and informed each other about upcoming events and staff visits. - Parental involvement - Having supportive and cooperative food service staff members. - Having food service managers and staff enjoy and be positive for trainings. - With support and enthusiasm from food service staff many of the guidelines could be implemented. | NR | - Lack of time to teach the program - Some activities were difficult to schedule and took away from other important curricula. - Lack of motivation from the teachers. - The curriculum being "too much work".  - Lack of support for program objectives. - Turnover in administration. - Scheduling conflicts. - Administration being too busy. - Lack of communication among staff and administration. - The program not being a top priority to administration. - Principals being too busy to get involved with events and did not show interest or enthusiasm for the program. - Lack of public endorsement - Lack of communication and support. |
| The diamond level health promoting schools (DLHPS) (31) | NR | - The obesity management programs succeeded due mainly to the school directors and the cooperation of all parties (teachers, parents, and students) - The participation of all stakeholders included public health representatives from the province, district, and sub-district; teachers; and representatives of community and parents - All school directors expressed their intentions to continue the programs in the future because these symbolised HPS | NR | - Teachers' confusion about the requirements in attaining the DLHPS - Workload of teachers and having to do too much paperwork |
| WAVE (32) | NR | - Having classroom lessons which give students the opportunity to practice skills rather than learn facts only - Meeting staff needs for professional development and curriculum support - Linking in with local Indigenous groups or tribes - Partnerships between schools and community - Intersectoral collaboration between health and education sectors - Having program facilitators - Having access to specialist health promotion expertise, Resource Centre, professional development and financial support from WAVE. | NR | - Lack of time for both parents and teachers - Transport in rural communities - Parent's belief that health promotion is the role of teachers and some parents' lack of confidence |
