# Supplementary material for: Applying the Integrated Sustainability Framework to explore the long-term sustainability of nutrition education programmes in schools: a systematic review
Source: Public Health Nutr. 2023 Aug 7;26(10):2165–79. doi: 10.1017/S1368980023001647 (PMC10564612; doi:10.1017/S1368980023001647)
Supplement: Supplementary file 1 [file S1368980023001647sup.zip › S1368980023001647sup004.docx]

**Supplementary Material 4: Detailed data analysis of extracted data using the Integrated Sustainability Framework**

| **Name of programme and references** | **Integrated Sustainability Framework domain and factors** | **Barriers to sustainment of health behaviour interventions** | **Enablers to sustainment of health behaviour interventions** |
| --- | --- | --- | --- |
| ***Action Schools! BC* (23,24)** | **Outer contextual factors** | | |
|  | Socio-political context | NR | Adopting and implementing the Provincial Guidelines for Food and Beverage Sales in the Schools. |
|  | Funding environment and availability | Costs not being reasonable. | Access to funding. |
|  | External partnerships and leadership/environmental support | NR | Community engagement  Connection with parents. |
|  | Values, needs and priorities | Yes  Inconsistent messages in the community | NR |
|  | **Inner contextual factors** | | |
|  | Programme champions | NR | Having a school champion |
|  | Organisational leadership/support | Lack of leadership caused by high staff turnover  Admin support. | Having a school leader |
|  | Organisational readiness/resources | Use was lower in schools where the principals agreed that the climate was supportive of HE. | Positive organisational climate  School commitment |
|  | Organisational stability | Lack of leadership caused by high staffing turnover | NR |
|  | **Processes** | | |
|  | Partnership/engagement | Lack of momentum. | The children enjoy it and that motivates me. |
|  | Training/supervision/support | Lack of resources (school equipment), leadership and momentum. | Support from the AS! BC central team.  Access to resources (high cost of kits).  Training and support (follow-ups) |
|  | Programme evaluation/data | Time to complete evaluation processes (logging) | NR |
|  | Adaptation | NR | NR |
|  | Communications and strategic planning | Lack of time to deliver the activities | Follow-up support |
|  | **Characteristics of the interventionists and population** | | |
|  | Implementer characteristics | NR | High self-efficacy |
|  | Implementer benefits and stressors | NR | NR |
|  | Implementer skills/expertise | Lack of knowledge of healthy living | Teachers who had healthy eating training. |
|  | Population characteristics | Student reaction (poor student engagement with program activities/resources). | NR |
|  | **Characteristics of the intervention** | | |
|  | Adaptability of EBI/fidelity | NR | Keeping the HE model relevant, fresh and responsive to teachers' needs |
|  | Fit with context/population/organisation | Larger schools were less likely to be users. | Integration within the curriculum.  Easy to use.  Schools in less urbanised areas. |
|  | Perceived benefits | NR | Positive impacts on children’s attitudes and behaviours. |
|  | Perceived need | NR | Top priority in their school.  Community need. |

| **Name of programme and references** | **Integrated Sustainability Framework domain and factors** | **Barriers to sustainment of health behaviour interventions** | **Enablers to sustainment of health behaviour interventions** |
| --- | --- | --- | --- |
| ***Crunch & Sip* (20,22)** | **Outer contextual factors** | | |
|  | Socio-political context | NR | NR |
|  | Funding environment and availability | NR | Sufficient money. |
|  | External partnerships and leadership/environmental support | NR | NR |
|  | Values, needs and priorities | NR | NR |
|  | **Inner contextual factors** | | |
|  | Programme champions | NR | NR |
|  | Organisational leadership/support | Lack of clarity and overlap of roles undertaken by NGO and LHS staff increased inconsistent delivery and decreased program efficiency. | NR |
|  | Organisational readiness/resources | Principals who agreed that implementing the program is far less important than other priorities within the school. | NR |
|  | Organisational stability | NR | NR |
|  | **Processes** | | |
|  | Partnership/engagement | Two-step delivery model (registration and certification). Teachers found the process “confusing”. | NR |
|  | Training/supervision/support | Minimal follow-up after certification had been received. | NR |
|  | Programme evaluation/data | NR | NR |
|  | Adaptation | NR | NR |
|  | Communications and strategic planning | NR | Sufficient resources to implement the program (time). |
|  | **Characteristics of the interventionists and population** | | |
|  | Implementer characteristics | NR | NR |
|  | Implementer benefits and stressors | NR | NR |
|  | Implementer skills/expertise | NR | NR |
|  | Population characteristics | NR | NR |
|  | **Characteristics of the intervention** | | |
|  | Adaptability of EBI/fidelity | NR | NR |
|  | Fit with context/population/organisation | That the program would be too difficult to implement (complexity of program). | The program needed to be simple to appeal to teaching staff. |
|  | Perceived benefits | NR | There is evidence that the program increases student consumption of veg and fruit. |
|  | Perceived need | NR | NR |

| **Name of programme and references** | **Integrated Sustainability Framework domain and factors** | **Barriers to sustainment of health behaviour interventions** | **Enablers to sustainment of health behaviour interventions** |
| --- | --- | --- | --- |
| ***Food Dudes* (26)** | **Outer contextual factors** | | |
|  | Socio-political context | NR | The presence of pre-existing whole school policies. |
|  | Funding environment and availability | NR | Having secured funding. Budgetary reduction. |
|  | External partnerships and leadership/environmental support | NR | Parental support, including time commitment, providing transport, and changes at home |
|  | Values, needs and priorities | NR | NR |
|  | **Inner contextual factors** | | |
|  | Programme champions | NR | NR |
|  | Organisational leadership/support | NR | NR |
|  | Organisational readiness/resources | Lack of canteens which provided logistical challenges.  Lack of support from additional staff and parents to sort out the fruit and vegetables. | NR |
|  | Organisational stability | NR | NR |
|  | **Processes** | | |
|  | Partnership/engagement | NR | NR |
|  | Training/supervision/support | NR | NR |
|  | Programme evaluation/data | Time required to implement and record evaluation and monitoring data.  Difficulty with monitoring lunchboxes. | NR |
|  | Adaptation | NR | NR |
|  | Communications and strategic planning | Insufficient communication between stakeholders at all levels.  Poor translation of research into practice (research staff not showing teachers how to implement the program thoroughly). | NR |
|  | **Characteristics of the interventionists and population** | | |
|  | Implementer characteristics | NR | NR |
|  | Implementer benefits and stressors | NR | NR |
|  | Implementer skills/expertise | NR | NR |
|  | Population characteristics | NR | NR |
|  | **Characteristics of the intervention** | | |
|  | Adaptability of EBI/fidelity | NR | Fidelity to core components.  Adaptations to FD were seen as enabling factors in ensuring successful implementation e.g. adaptations to program delivery were made for children with special needs and/or serious aversions to specific fruit or vegetables and the timing of the intervention was customised to suit the school timetable.  Simplicity of intervention. |
|  | Fit with context/population/organisation | Time constraints to deliver prescribed curriculum.  SUSTAINABILITY  If the program is not embedded in an organisational structure which offered support to the program e.g. pre-existing healthy eating policies, which reflected the ethos and commitment of the school. | SUSTAINABILITY  Having the program embedded in an organisational structure which offered support to the program e.g. pre-existing healthy eating policies, which reflected the ethos and commitment of the school. |
|  | Perceived benefits | NR | Incentives and rewards for the children |
|  | Perceived need | NR | NR |

| **Name of programme and references** | **Integrated Sustainability Framework domain and factors** | **Barriers to sustainment of health behaviour interventions** | **Enablers to sustainment of health behaviour interventions** |
| --- | --- | --- | --- |
| ***Food for Fitness* (27)** | **Outer contextual factors** | | |
|  | Socio-political context | NR | NR |
|  | Funding environment and availability | NR | NR |
|  | External partnerships and leadership/environmental support | NR | Parental support to take the lessons home. |
|  | Values, needs and priorities | NR | NR |
|  | **Inner contextual factors** | | |
|  | Programme champions | NR | NR |
|  | Organisational leadership/support | SUSTAINABILITY  Lack of extended school support. | NR |
|  | Organisational readiness/resources | Difficulties regarding class organisation and timing (activities going over-time). | Limited staff and facilities as a restrictive capacity issue. |
|  | Organisational stability | NR | NR |
|  | **Processes** | | |
|  | Partnership/engagement | NR | NR |
|  | Training/supervision/support | Need for ongoing support for management of the delivery of content, particularly timing of engagement, availability, accessibility. | Clear consistent evidence-based messages in training and advice.  Training and supporting the teachers or anyone who delivers a key skill. |
|  | Programme evaluation/data | NR | NR |
|  | Adaptation | NR | NR |
|  | Communications and strategic planning | Poor class organisation and timing.  Inefficient planning processes. | NR |
|  | **Characteristics of the interventionists and population** | | |
|  | Implementer characteristics | NR | NR |
|  | Implementer benefits and stressors | NR | NR |
|  | Implementer skills/expertise | NR | Using skilled and knowledgeable staff with a practical and applied approach.  Recognition of the multiple learning styles involved in the delivery of lessons. |
|  | Population characteristics | NR | NR |
|  | **Characteristics of the intervention** | | |
|  | Adaptability of EBI/fidelity | NR | NR |
|  | Fit with context/population/organisation | NR | Program should be acceptable for the school environment.  Complementing other school-based programs.  Being easily embedded into existing curriculum. |
|  | Perceived benefits | NR | Adds value when no existing food policies are in place at the school.  Opportunities to improve social skills and life skills. |
|  | Perceived need | NR | NR |

| **Name of programme and references** | **Integrated Sustainability Framework domain and factors** | **Barriers to sustainment of health behaviour interventions** | **Enablers to sustainment of health behaviour interventions** |
| --- | --- | --- | --- |
| ***Health Promoting Schools (HPS)* (25)** | **Outer contextual factors** | | |
|  | Socio-political context | Conflicting policy (between HPS and school/district policies). | High level policy and institutional anchoring of health-promoting schools (HPS). |
|  | Funding environment and availability | NR | NR |
|  | External partnerships and leadership/environmental support | Limited engagement in community partnerships. | Support provided by community partners. |
|  | Values, needs and priorities | Conflicting priorities policy (between HPS and school/district priorities). | HPS philosophy shared by their respective school districts. |
|  | **Inner contextual factors** | | |
|  | Programme champions | NR | NR |
|  | Organisational leadership/support | Insufficient staff and volunteer capacity. | Supportive leadership from the principal who also provided flexibility to enable support among school staff.  Distributed leadership through shared responsibilities for activity implementation.  Support provided by committed school staff.  Having a committee or team that meets to plan activities.  Collaborative engagement.  Leadership and management practices. |
|  | Organisational readiness/resources | Limited organisational capacity and time.  Health promotion not being a school priority.  Limited dedicated resources. | NR |
|  | Organisational stability | NR | Relational and organisational context: staff behaviours and collaboration. |
|  | **Processes** | | |
|  | Partnership/engagement | Limited engagement in community partnerships. | School partnerships and networking. |
|  | Training/supervision/support | NR | Professional developing and learning. |
|  | Programme evaluation/data | NR | NR |
|  | Adaptation | NR | NR |
|  | Communications and strategic planning | NR | Preparing and planning for school development: policies, leadership, goals, practices, school planning. |
|  | **Characteristics of the interventionists and population** | | |
|  | Implementer characteristics | NR | NR |
|  | Implementer benefits and stressors | NR | NR |
|  | Implementer skills/expertise | NR | NR |
|  | Population characteristics | NR | NR |
|  | **Characteristics of the intervention** | | |
|  | Adaptability of EBI/fidelity | NR | NR |
|  | Fit with context/population/organisation | SUSTAINABILITY  Educational and cultural priorities limiting health promotion and sustainability.  Challenging to balance health promotion initiatives with current academic priorities. | Student-centred learning, an emphasis on skill development and fostering enjoyment among students. Student participation and engagement.  Culture for health promotion. Integration of health into priorities and the culture of the school. |
|  | Perceived benefits | NR | NR |
|  | Perceived need | NR | NR |

| **Name of programme and references** | **Integrated Sustainability Framework domain and factors** | **Barriers to sustainment of health behaviour interventions** | **Enablers to sustainment of health behaviour interventions** |
| --- | --- | --- | --- |
| ***Healthy Choices* (30)** | **Outer contextual factors** | | |
|  | Socio-political context | NR | NR |
|  | Funding environment and availability | Budget limitations make it difficult to make changes in dining services. | SUSTAINABILITY  Importance of future funding. |
|  | External partnerships and leadership/environmental support |  | SUSTAINABILITY  Necessity of parental/community support. |
|  | Values, needs and priorities | Time constraints owing to state-mandated testing. | SUSTAINABILITY  Reducing MCAS (state-mandated testing) related pressures. |
|  | **Inner contextual factors** | | |
|  | Programme champions | NR | Having a program champion. |
|  | Organisational leadership/support | NR | SUSTAIINABILITY  Continued buy-in and support from administration and staff (maintaining existing supports while acquiring additional ones).  Employing a team approach.  Administrative and staff buy-in. |
|  | Organisational readiness/resources | General time constraints. | NR |
|  | Organisational stability | NR | NR |
|  | **Processes** | | |
|  | Partnership/engagement | NR | SUSTAINABILITY  Maintaining contact with outside experts.  School coordinator’s string relationships with faculty and staff. |
|  | Training/supervision/support | Difficulty in training teachers in Planet Health. | External support offered (e.g. regional coordinators, program trainings). |
|  | Programme evaluation/data | NR | NR |
|  | Adaptation | NR | NR |
|  | Communications and strategic planning | NR | NR |
|  | **Characteristics of the interventionists and population** | | |
|  | Implementer characteristics | Unwillingness of some teachers to use Planet Health.  Perceived reluctance of food service personnel to make changes. | NR |
|  | Implementer benefits and stressors | NR | NR |
|  | Implementer skills/expertise | NR | NR |
|  | Population characteristics | NR | NR |
|  | **Characteristics of the intervention** | | |
|  | Adaptability of EBI/fidelity | NR | NR |
|  | Fit with context/population/organisation | NR | NR |
|  | Perceived benefits | NR | NR |
|  | Perceived need | NR | NR |

| **Name of programme and references** | **Integrated Sustainability Framework domain and factors** | **Barriers to sustainment of health behaviour interventions** | **Enablers to sustainment of health behaviour interventions** |
| --- | --- | --- | --- |
| ***KEIGAAF* (21)** | **Outer contextual factors** | | |
|  | Socio-political context | NR | National health promoting trends. The guidelines of the national HPS committee concerning the HPS certificates enabled all schools to set priorities. Other national trends, such as the EU school fruit program or national initiatives such as National Sports Week, also facilitated the schools in the implementation of PA and healthy eating promotion. |
|  | Funding environment and availability | NR | Financial support |
|  | External partnerships and leadership/environmental support | Lack of potential partners. A lack of potential partners or limited collaborations between school and potential partners hindered the implementation of comprehensive PA and healthy nutrition promotion.  Low parental support and involvement with school activities (whether health-related or not). | Practical support of health promoting advisors. Some working groups required guidance and encouragement of the HP advisor. The HP advisor sought the best strategy to guide and encourage a working group. A good match between these strategies and the needs of the working group facilitated implementation.  Top-down influence from school board. The school board's demand to obtain the HPS certificates supported the KEIGAAF approach by accelerating the implementation of PA and nutrition-promoting activities. |
|  | Values, needs and priorities | NR | NR |
|  | **Inner contextual factors** | | |
|  | Programme champions | NR | Champion. A factor facilitating the integration of the activities within a school was the presence of a champion. |
|  | Organisational leadership/support | Nutrition as intervention topic was a barrier to implementation due to lack of support from multiple actors. | Support of school staff, principal and parents. School staff support facilitated the integration of activities and policies within the school. Schools that were most active in implementation had a principal who supported the working group, agreed on decisions made by the working group, demanded that the rest of the school staff support the implementation of this plan. |
|  | Organisational readiness/resources | Starting situation school health promotion. When the working group considered that their school was already making much effort regarding PA and nutrition promotion, implementation was hindered. | A good starting situation school health promotion- the working group considered that the current situation had to change (limited PA or healthy nutrition promotion at the school). |
|  | Organisational stability | At some schools high employee turnover inhibited implementation because of poor communication between the leaving employee(s) and the new employee(s) or uncertainty about division of tasks. | Employee turnover. In most schools, the employee turnover facilitated the implementation of the intervention, because the new members were more practice, had more decisional power, or because the changes could be more easuly implemented given the teachers' unfamiliarity with the old practices. |
|  | **Processes** | | |
|  | Partnership/engagement | NR | NR |
|  | Training/supervision/support | NR | NR |
|  | Programme evaluation/data | Feedback loops. The results of the behavioural measurements of the children were demotivating for some schools because, in the short term, the children did not improve in their behavioural outcomes. | Feedback loops in the intervention supported implementation. The timeline sessions were very helpful in deciding on or improving their activity plans. |
|  | Adaptation | NR | NR |
|  | Communications and strategic planning | SUSTAINABILITY  Practice orientated thinking of the working groups did not facilitate the development of a deliberated and sustainable plan.  Misinterpretation of the intervention approach and the intervention objectives inhibited implementation. | Positive dynamics. Good interaction between working group members, including constructive communication between he working group members and the HP advisor, facilitated the process of developing and implementing.  Practice-orientated thinking of the working groups facilitated implementation |
|  | **Characteristics of the interventionists and population** | | |
|  | Implementer characteristics | NR | NR |
|  | Implementer benefits and stressors | NR | NR |
|  | Implementer skills/expertise | NR | NR |
|  | Population characteristics | NR | NR |
|  | **Characteristics of the intervention** | | |
|  | Adaptability of EBI/fidelity | NR | SUSTAINABILITY  Intervention being characterised by a high degree of adaptation and local tailoring.  Adaptation. The KEIGAAF intervention was characterised by a high degree of adaptation and local tailoring which facilitated the implementation of activities that were suitable for the local context. It also enhanced feelings of ownership and sustainability of the bottom-up approach. |
|  | Fit with context/population/organisation | NR | NR |
|  | Perceived benefits | NR | NR |
|  | Perceived need | NR | NR |

| **Name of programme and references** | **Integrated Sustainability Framework domain and factors** | **Barriers to sustainment of health behaviour interventions** | **Enablers to sustainment of health behaviour interventions** |
| --- | --- | --- | --- |
| ***New Moves* (28)** | **Outer contextual factors** | | |
|  | Socio-political context | NR | NR |
|  | Funding environment and availability | Lack of adequate finances was a reason that teachers did not offer guest instructors or hold weekly lunch get togethers. | Requires minimal additional money outside of the classroom to implement the program. |
|  | External partnerships and leadership/environmental support | NR | NR |
|  | Values, needs and priorities | NR | NR |
|  | **Inner contextual factors** | | |
|  | Programme champions | NR | NR |
|  | Organisational leadership/support | NR | SUSTAINABILITY  Feelings of support and actual support for the class by school administrators.  Receive buy-in and support from other teachers/staff. |
|  | Organisational readiness/resources | Lack of adequate time for teachers to meet with students outside of class for individual counselling meetings. | Require minimal additional time outside of the classroom to implement the program. |
|  | Organisational stability | Teacher turnover (due to lack of training, some teachers not having received materials and lack of ongoing ownership of the intervention. | NR |
|  | **Processes** | | |
|  | Partnership/engagement | NR | NR |
|  | Training/supervision/support | NR | SUSTAINABILITY  Receiving teacher training on the New Moves program from the research team. |
|  | Programme evaluation/data | NR | NR |
|  | Adaptation | NR | NR |
|  | Communications and strategic planning | NR | NR |
|  | **Characteristics of the interventionists and population** | | |
|  | Implementer characteristics | NR | NR |
|  | Implementer benefits and stressors | NR | SUSTAINABILITY  Teachers believing in the importance of the program.  Teachers who were motivated to continue the class. |
|  | Implementer skills/expertise | NR | NR |
|  | Population characteristics | Low motivation among participants. | NR |
|  | **Characteristics of the intervention** | | |
|  | Adaptability of EBI/fidelity | NR | NR |
|  | Fit with context/population/organisation | Class size (too large or too small). Large class sizes led to difficulties in class management, whereas class sizes that were too small were not sustainable due to school policies for minimal class sizes.  Fit into current school structure. | Teachers who had worked with counsellors and who had taken an active role in determining their class list were ore successful at overcoming large class sizes. |
|  | Perceived benefits | NR | Teachers perceived increased confidence and greater participation of the students (higher teacher and student satisfaction). |
|  | Perceived need | NR | NR |

| **Name of programme and references** | **Integrated Sustainability Framework domain and factors** | **Barriers to sustainment of health behaviour interventions** | **Enablers to sustainment of health behaviour interventions** |
| --- | --- | --- | --- |
| ***Pathways* (29)** | **Outer contextual factors** | | |
|  | Socio-political context | NR | NR |
|  | Funding environment and availability | NR | NR |
|  | External partnerships and leadership/environmental support | Lack of public endorsement | Parental involvement. |
|  | Values, needs and priorities | NR | NR |
|  | **Inner contextual factors** | | |
|  | Programme champions | NR | NR |
|  | Organisational leadership/support | Lack of support from teachers for program objectives.  The program not being a top priority to administration.  Principals being too busy to get involved with events and did not show interest or enthusiasm for the program.  Pressure from the school board to not spend as much time on the program. | Teachers who worked together and supported one another.  Having supportive and cooperative food service staff members. With support and enthusiasm from food service staff many of the guidelines could be implemented. |
|  | Organisational readiness/resources | Lack of time to prepare for and teach the program. Administration being too busy. | Positive school climate. |
|  | Organisational stability | Turnover in administration (being indicative of poor organisational climate). No continuity with involvement or limited interest from new administration staff. | NR |
|  | **Processes** | | |
|  | Partnership/engagement | NR | NR |
|  | Training/supervision/support | NR | Having food service managers and staff enjoy and be positive for trainings. |
|  | Programme evaluation/data | NR | NR |
|  | Adaptation | NR | NR |
|  | Communications and strategic planning | Scheduling conflicts.  Lack of communication between staff and administration. | Good communication and support when the administration and teachers worked together and informed each other about upcoming events and staff visits. |
|  | **Characteristics of the interventionists and population** | | |
|  | Implementer characteristics | Lack of motivation from the teachers. | Having committed teachers was seen as very important to the overall involvement of parents, children and administration.  Teachers being role models to the students. |
|  | Implementer benefits and stressors | NR | NR |
|  | Implementer skills/expertise | NR | NR |
|  | Population characteristics | NR | NR |
|  | **Characteristics of the intervention** | | |
|  | Adaptability of EBI/fidelity | NR | NR |
|  | Fit with context/population/organisation | The curriculum being "too much work".  Some activities were difficult to schedule and took away from other important curricula. | NR |
|  | Perceived benefits | NR | The program teaching family members about healthy eating and physical activity.  Parents becoming more involved with the child’s school. |
|  | Perceived need | NR | NR |

| **Name of programme and references** | **Integrated Sustainability Framework domain and factors** | **Barriers to sustainment of health behaviour interventions** | **Enablers to sustainment of health behaviour interventions** |
| --- | --- | --- | --- |
| ***DLHPS* (31)** | **Outer contextual factors** | | |
|  | Socio-political context | NR | NR |
|  | Funding environment and availability | NR | NR |
|  | External partnerships and leadership/environmental support | Lack of parental support. | The participation of all stakeholders included public health representatives from the province, district, and sub-district; teachers; and representatives of community and parents.  Parents being positive about healthy behaviours at home. |
|  | Values, needs and priorities | NR | All school directors expressed their intentions to continue the programs in the future because these symbolised HPS |
|  | **Inner contextual factors** | | |
|  | Programme champions | NR | NR |
|  | Organisational leadership/support | NR | The obesity management programs succeeded due mainly to the school directors and the cooperation of all parties (teachers, parents, and students) |
|  | Organisational readiness/resources | Workload of teachers. | NR |
|  | Organisational stability | NR | NR |
|  | **Processes** | | |
|  | Partnership/engagement | NR | NR |
|  | Training/supervision/support | Lack of support and training to help teachers understand the requirements in attaining the DLHPS. | Having public health staff explain to teachers how to attain the DLHPS and how to fill out the associated document. |
|  | Programme evaluation/data | Having to do too much paper work (additional workload). | NR |
|  | Adaptation | NR | NR |
|  | Communications and strategic planning | NR | Dissemination of health information to parents. |
|  | **Characteristics of the interventionists and population** | | |
|  | Implementer characteristics | NR | NR |
|  | Implementer benefits and stressors | NR | NR |
|  | Implementer skills/expertise | NR | NR |
|  | Population characteristics | NR | NR |
|  | **Characteristics of the intervention** | | |
|  | Adaptability of EBI/fidelity | NR | New activities to add to the program to increase its effectiveness. |
|  | Fit with context/population/organisation | NR | NR |
|  | Perceived benefits | NR | NR |
|  | Perceived need | NR | NR |

| **Name of programme and references** | **Integrated Sustainability Framework domain and factors** | **Barriers to sustainment of health behaviour interventions** | **Enablers to sustainment of health behaviour interventions** |
| --- | --- | --- | --- |
| ***WAVE* (32)** | **Outer contextual factors** | | |
|  | Socio-political context | NR | NR |
|  | Funding environment and availability | NR | NR |
|  | External partnerships and leadership/environmental support | NR | Linking in with local Indigenous groups or tribes.  Partnerships between schools and community.  Intersectoral collaboration between health and education sectors |
|  | Values, needs and priorities | Parent's belief that health promotion is the role of teachers and some parents' lack of confidence | NR |
|  | **Inner contextual factors** | | |
|  | Programme champions | NR | NR |
|  | Organisational leadership/support | NR | NR |
|  | Organisational readiness/resources | Lack of time for teachers. | NR |
|  | Organisational stability | NR | NR |
|  | **Processes** | | |
|  | Partnership/engagement | NR | Having program facilitators. |
|  | Training/supervision/support | NR | Meeting staff needs for professional development and curriculum support.  Having access to specialist health promotion expertise, Resource Centre, professional development and financial support from WAVE. |
|  | Programme evaluation/data | NR | NR |
|  | Adaptation | NR | NR |
|  | Communications and strategic planning | NR | NR |
|  | **Characteristics of the interventionists and population** | | |
|  | Implementer characteristics | NR | NR |
|  | Implementer benefits and stressors | NR | NR |
|  | Implementer skills/expertise | NR | NR |
|  | Population characteristics | NR | NR |
|  | **Characteristics of the intervention** | | |
|  | Adaptability of EBI/fidelity | NR | NR |
|  | Fit with context/population/organisation | Transport in rural communities. | Having classroom lessons which give students the opportunity to practice skills rather than learn facts only |
|  | Perceived benefits | NR | NR |
|  | Perceived need | NR | NR |
